# The development of the human notochord

**DOI:** 10.1371/journal.pone.0205752

**Published:** 2018-10-22

**Authors:** Karel de Bree, Bernadette S. de Bakker, Roelof-Jan Oostra

**Affiliations:** Department of Medical Biology, Section Clinical Anatomy & Embryology, Amsterdam UMC, University of Amsterdam, Amsterdam, The Netherlands; University of Colorado Boulder, UNITED STATES

## Abstract

The notochord is a major regulator of embryonic patterning in vertebrates and abnormal notochordal development is associated with a variety of birth defects in man. Proper knowledge of the development of the human notochord, therefore, is important to understand the pathogenesis of these birth defects. Textbook descriptions vary significantly and seem to be derived from both human and animal data whereas the lack of references hampers verification of the presented data. Therefore, a verifiable and comprehensive description of the development of the human notochord is needed. Our analysis and three-dimensional (3D) reconstructions of 27 sectioned human embryos ranging from Carnegie Stage 8 to 15 (17–41 days of development), resulted in a comprehensive and verifiable new model of notochordal development. Subsequent to gastrulation, a transient group of cells briefly persists as the *notochordal process* which is incorporated into the endodermal roof of the gut while its dorsal side attaches to the developing neural tube. Then, the *notochordal process* embeds entirely into the endoderm, forming the epithelial *notochordal plate*, which remains intimately associated with the neural tube. Subsequently, the notochordal cells detach from the endoderm to form the definitive notochord, allowing the paired dorsal aortae to fuse between the notochord and the gut. We show that the formation of the *notochordal process* and *plate* proceeds in cranio-caudal direction. Moreover, in contrast to descriptions in the modern textbooks, we report that the formation of the definitive notochord in humans starts in the middle of the embryo, and proceeds in both cranial and caudal directions.

## Introduction

The definitive notochord is a rod-like structure situated ventral to the neural tube in vertebrate embryos. When the body plan is laid down the notochord is crucial for the maintenance of the left-right asymmetry and it has an important inductive and regulatory role among adjunct tissues in early vertebrate development [1, 2]. It is such an evolutionary well-preserved structure that the Chordates, a group of animals that includes Tunicates like sea squirts, Cephalochordates like lancelets and Vertebrates like humans, are named after the notochord.

Abnormal notochordal development results in malformations of the gut, neural tube, vertebrae and cranial region [3–8]. A specific kind of aggressive neuraxial tumors, called chordomas, seems to develop from ectopic remnants of the notochord and thus can be considered to result also from abnormal notochordal development [9–11]. In 2014, Postma, *et al*. identified three human families with a persistent *notochordal canal*, due to a mutation in the brachyury gene, which encodes a crucial gene in the pathway of axial mesoderm differentiation [12]. Insight into the origin and clinical effects of these abnormalities requires knowledge of the normal development of the human notochord, which, therefore, is of great clinical interest.

**Fig 1:**
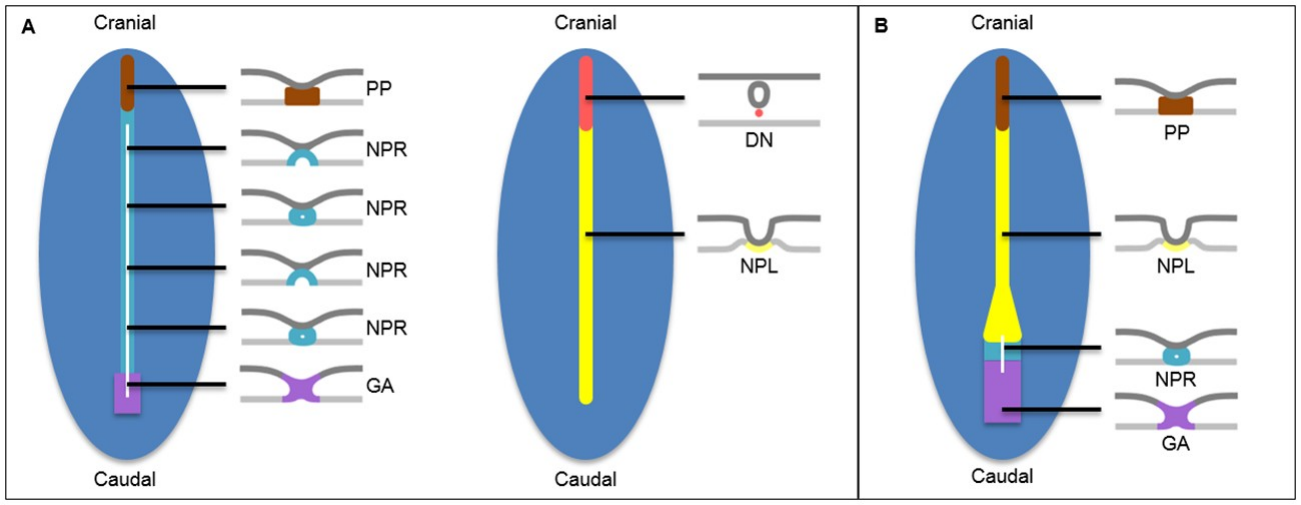
Human notochord development textbook concepts compared to our proposed theory. Schematic summary of the two main developmental concepts in modern textbooks. Ventral view with removed endoderm. The black lines indicate the level of the transversal sections (epiblast/ectoderm is superior in each section). In **A** the notochord develops in two phases. First the *notochordal process* (NP) extends over almost the entire length of the embryo (left). Within the *notochordal process* is a luminal extension of the primitive pit. The NP intercalates between the endoderm and the bottom of the NP ‘breaks down’ along the length of the *neurenteric canal*. This process results in the *notochordal plate* (right), which retracts between the mesenchyme forming the *definitive notochord*. **B** Illustrates our proposed theory. All precursors of the notochord are simultaneously present. We now know that the NP is shorter in length and that the definitive notochord does not develop cranially in the embryo, but merely in the middle region (Figs 3 and 5). *Definitive notochord* (DN): red, endoderm: light grey (in sections), epiblast or ectoderm: dark blue, dark grey (in sections), gastrulation area (GA): purple, *neurenteric canal*: white, *notochordal plate* (NPL): yellow, *notochordal process* (NPR): cyan blue, *prechordal plate* (PP): brown.

A century of research on the development of the notochord has not resulted in uniform representations of this process in medical textbooks underscored by appropriate references [13–19]. This may indicate that the morphological process is not clear. Apart from discrepancies in time of appearance, reported differences relate to the position of the first notochordal cells, the so-called *notochordal process*. Most authors position the *notochordal process* in-between the endoderm and ectoderm, which is the position of the definitive notochord. These cells would then become incorporated into the endoderm, forming the *notochordal plate* and subsequently would detach from the endoderm to form the definitive notochord. Other authors position the *notochordal process* directly embedded in the endodermal germ layer and then follow the same process (Fig 1A) [13–19]. Cartoons showing such a transient structure sometimes encompass the entire longitudinal axis, obviously at odds with the transient nature of such a structure.

Be that as it may, the ambiguities in present-day textbooks on the spatiotemporal development of the human notochord and current literature on experimental animals justifiably raise the question as to the basis of current descriptions of the development of the notochord in human.

This study describes the development of the human notochord based on histological sections of human embryos and three-dimensional (3D) reconstructions [20]. The 3D visualization enables the explicit analysis of the notochord in relation to its neighbouring structures. This provides a verifiable and comprehensible overview of human notochordal development, which occurs from Carnegie stages 7 to 12 (15–30 days of development).

## Materials and methods

Human embryos are staged according to the Carnegie staging criteria [21–25]. These stages are determined by external features that are present at that particular stage [21]. To study the development of the notochord, 23 human embryos from Carnegie stage 7 up to and including stage 12 were analysed by both histological sections and 3D reconstructions (Table 1). In addition, two stage 13 (28–32 days), 14 (31–35 days) and 15 (41 days) human embryos were used to analyze the development of the notochord in relation to the fusion of the paired dorsal aortae.

**Table 1:** An overview of the specimens used for the atlas with the available information [21–28].

CS	Specimen #	Origin^a^	Year	Acquired through	CRL (mm)	Day	Fixation medium	Staining	Plane^b^	Z-res (μm)
7	7802	CC	1940	Hysterectomy	0.42	19	Bouin	Hematoxylin and Eosin	o	10.00
7	8752	CC	1950	Hysterectomy	0.43		Unknown	Hematoxylin and Eosin	o	6.00
8	5960	CC	1929	Hysterectomy	1.52	18	Kaiserling	Al. Coch. & eosin	o	5.00
8	7545	CC	1938	No information	Unknown		Formol	Hematoxylin and Eosin	t	6.00
8	7568	CC	1938	No information	Unknown		Formol	Al. Coch.	t	10.00
8	7972	CC	1942	No information	Unknown		Alcohol & Bouin	Hematoxylin and Eosin	s	6.00
8	8671	CC	1949	Hysterectomy	0.61		Alcohol & Bouin	Hematoxylin and Eosin	t	2.70
8	10157	CC	1967	Hysterectomy	1.16	23	Formol	Cason	t	5.34
9	1878	CC	1907	No information	1.38		Formol	Hematoxylin and Eosin	t	10.00
9	3709	CC	1921	No information	1.38	25	Formol	Erythrosin	t	9.08
9	5080	CC	1926	No information	1.50		Formol	Al. Coch.	t	10.00
9	H712	BC	1957	Hysterectomy	1.57		Formalin and Bouin	Hematoxylin and Eosin	t	3.59
9	N509	HDBR	2011	Abortion	2.40		Paraformaldehyde	Hematoxylin and Eosin	t	5.00
10	0391	CC	1907	No information	2.00		Formol	Al. Coch	t	10.00
10	3707	CC	1921	No information	1.50		Formol	I.H.	o	12.50
10	3710	CC	1921	No information	3.60		Formol	H. & or. G.	o	10.00
10	4216	CC	1923	No information	2.00		Formol	Unknown	o	15.00
10	5074	CC	1925	Abortion (EUG)	1.41		Bouin	Alum cochineal (i.e. carmine)	t	4.69
10	6330	CC	1931	No information	1.95	28	Formol	Ehrlich’s acid hematoxylin	t	11.63
11	6344	CC	1931	Hysterectomy	2.58	29	Formalin	Alum cochineal (i.e. carmine)	t	18.83
11	6784	CC	1933	Hysterectomy	2.46		Formol	Iron Hematoxylin	t	8.70
12	8505A	CC	1947	Miscarriage	2.86		Formol	Hematoxylin and Phloxin	t	10.32
12	8943	CC	1934	Hysterectomy	3.58		Zenker's Formol	Hematoxylin and Eosin	t	8.22
13	836	CC	1914	Hysterectomy	4.09	32	Mercuric Chlorine	Alum cochineal (i.e. carmine)	t	16.55
13	5541	CC	1927	Miscarriage	4.08	38	Formol	Alum cochineal, eosin	t	10.76
14	6502	CC	1931	No information	5.54		Could be Souza	Hematoxylin and Eosin	t	5.01
14	8314	CC	1945	Hysterectomy	5.16	22	Formol	Azan	t	8.07
15	721	CC	1913	No information	4.79	36	Zenker's Formol	Hematoxylin and Eosin	t	8.69
15	3512	CC	1921	Miscarriage	6.55		Formol	Alum cochineal (i.e. carmine)	t	10.06

CS: Carnegie stage, Year: Year of acquisition, CRL: Calculated crown-rump-length in mm, Day: days post ovulation, Z-res: Calculated Z-resolution in μm.

^a^ Origin of the specimen: CC = Carnegie Collection: Human Developmental Anatomy Center at the National Museum of Health and Medicine in Silver Spring, Maryland, USA // BC = Boyd Collection: Department of Physiology, Development and Neuroscience, University of Cambridge, United Kingdom // HDBR = Human Developmental Biology Resource, Institute of Genetic Medicine, International Centre for Life, Newcastle Upon Tyne, United Kindom.

^b^ Plane of sectioning: o = oblique, t = transversal, c = coronal

The embryos included in this study (Table 1) are historical specimens, originating from the Carnegie collection of the Developmental Anatomy Center at the National Museum of Health and Medicine in Silver Spring, MD, USA. One embryo originated from the Boyd Collection from the Department of Physiology, Development and Neuroscience, University of Cambridge, United Kingdom and one embryo was kindly donated by the Human Developmental Biology Resource, Institute of Genetic Medicine, International Centre for Life, Newcastle Upon Tyne, United Kingdom. No ethical approval was needed for this retrospective study.

The embryos had been sectioned in transversal or sagittal planes (Table 1). The histological sections were digitalized by acquiring high-resolution images with a digital camera attached to a microscope as described previously by de Bakker *et al*. [20]. When more than two embryos of a stage were available, the two embryos with the best histological quality were manually reconstructed using the method of de Bakker *et al*. [20]. The software package Amira 5.4 (http://www.amira.com) was used to create the 3D reconstructions.

## Results

**Fig 2:**
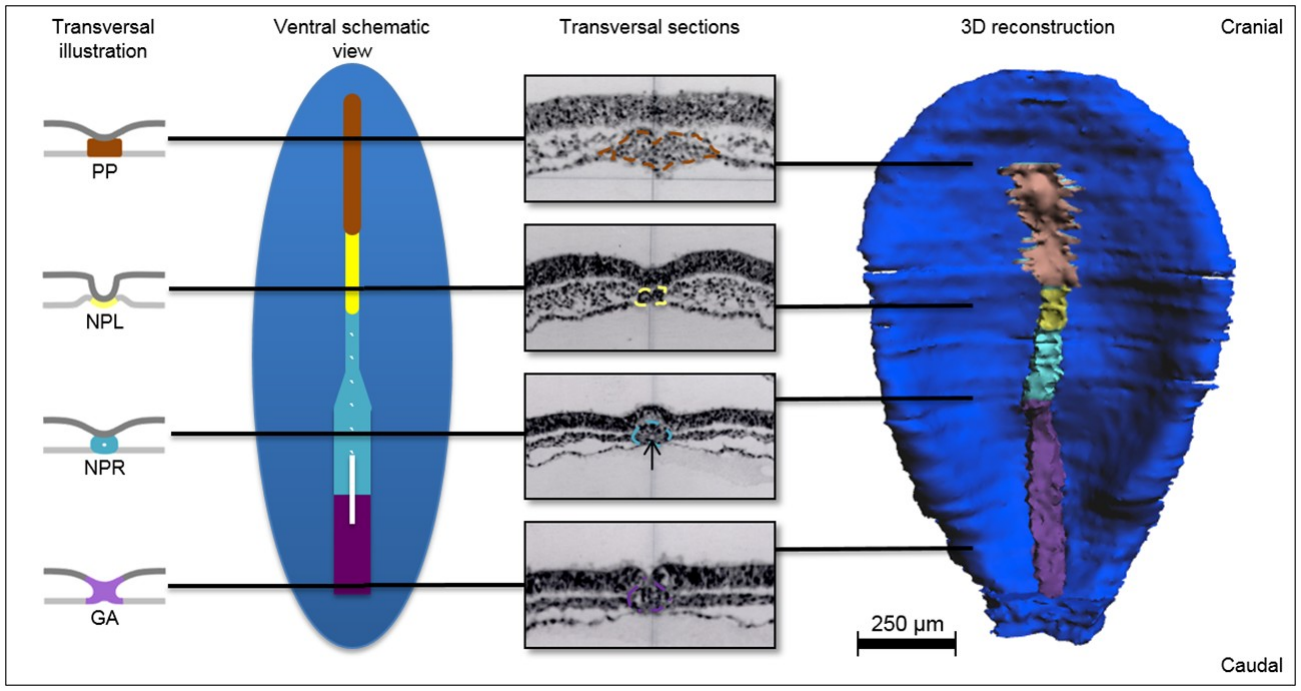
Development of the notochord in a stage 8 (17–19 days) human embryo. From left to right transversal illustrations, a schematic ventral view, transversal sections from specimen No. 7545 and a ventral view of a 3D reconstruction of specimen No. 5960. The embryo is viewed from ventral with the endoderm removed. Black lines indicate the level of the transversal sections (epiblast/ectoderm is superior in each transversal section). Subsequent to the gastrulation area (purple) the *notochordal process* (cyan blue) with a *neurenteric canal* (white on the schematic view and black arrow in the transversal sections) is present. The *notochordal process* is round, but further cranial it becomes smaller and the endoderm on its ventral side is hard to distinguish. Cranial in the embryo, the *prechordal plate* (brown) emerges as a cluster of mesenchymal cells. The *prechoral plate* can be identified in the 3D reconstruction as a broader structure cranial to the *notochordal plate*. Endoderm: light grey (in cross sections), epiblast or ectoderm: dark blue, dark grey (in transversal sections), gastrulation area (GA): purple, *neurenteric canal*: white, *notochordal plate* (NPL): yellow, *notochordal process* (NPR): cyan blue, *prechordal plate* (PP): brown.

**Fig 3:**
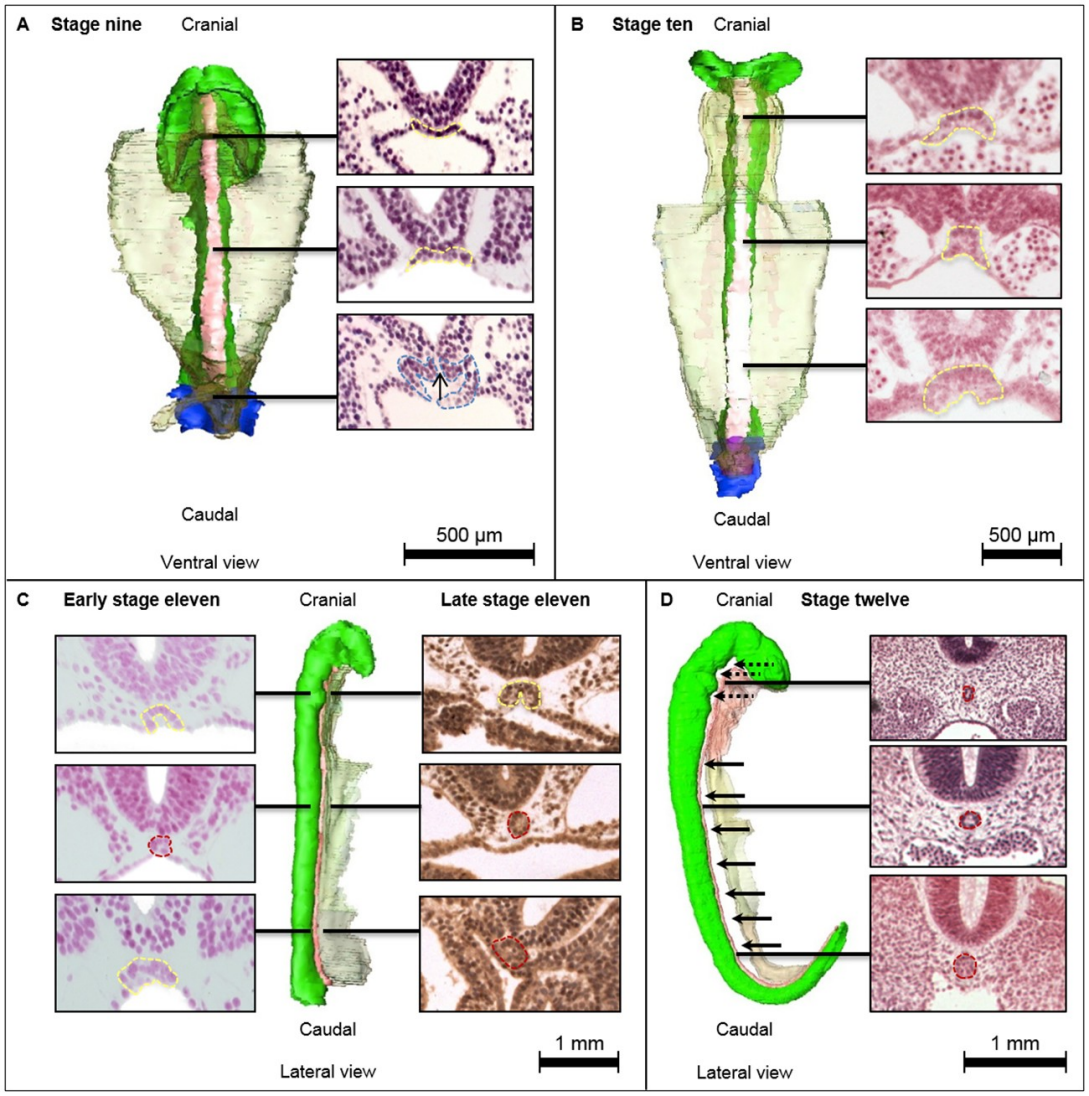
Development of the notochord in CS9 to CS12 (19–30 days). 3D reconstructions of specimens Nos. 3709, 6330, 6344, and 8943. Black lines indicate the level of the transversal histological sections (neural ectoderm is superior in each section). **A** CS9 (19-21d.) embryo with *notochordal plate* tightly attached to the neural tube on both lateral sides, forming a flat or U-shaped *notochordal plate* around the developing neural tube. Caudally a *notochordal process* with a *neurenteric canal*, indicated with a black arrow. **B** CS10 (21-23d.) embryo, the notochordal ridges are now pointing ventral, giving the notochordal plate the inverted U-shape. **C** Left an early CS11 (23-26d.) embryo (sections of the reconstruction) with a *definitive notochord* in the middle of the embryo and *notochordal plate* on both cranial and caudal extremes. The late CS11 embryo (right) specimen No. 6344 has a definitive notochord at the caudal extreme and in the middle. **D** In CS12 (26-30d.) all sections show a definite notochord. The caudal part of the notochord has a larger diameter. Note the space between notochord and endoderm (black arrows) and between notochord and neural tube (dotted arrows). Endoderm: transparent green, gastrulation: purple, neural ectoderm: green, *notochordal plate*: yellow, *Notochordal process*: cyan blue.

The development of the human notochord has three distinct phases, leading to the *definitive notochord*, also dubbed the *notochord proper* or *notochord senso stricto*. This structure develops in three phases between Carnegie stage 8 (17–19 days) and 12 (26–30 days). These phases are characterized by the presence of (1) the *notochordal process* and *prechordal plate* (or *prochordal plate*), (2) the *notochordal plate* and (3) the *definitive notochord*. The description of these structures and their appearance during human development are presented below in histological sections and 3D models (Figs 2 and 3). Three general observations on the development of the notochord are important to note.

Firstly, the developing notochord is always tightly attached to the ventral floor of the developing central nervous system. This is the case from its first appearance in stage 8 (17–19 days) until the *definitive notochord* is formed in stage 12 (26–30 days). Secondly, the middle region of the *notochordal plate* remains remarkably smaller compared to both the cranial and caudal extremes during stage 9 to 11 (19–26 days). Finally, the precursors of the definitive notochord develop in a craniocaudal fashion from stage 8 to 10 (17–23 days). However, from stage 10 to 12 (23–30 days) onward the *definitive notochord* establishes its final shape in a bidirectional course, from the middle of the embryo to both cranial and caudal directions.

### First appearance of the developing notochord; The notochordal process

According to the Carnegie staging criteria, the primordium of the notochord is first seen in stage 7 (15–17 days) embryos as the *notochordal process* [21]. In specimens No. 7802 and No. 8572 of the Carnegie collection, the process of gastrulation is still ongoing. However, neither a *notochordal process* nor a *prechordal plate* were observed. Because these specimens do not have a *notochordal process*, they should be considered as stage 6 embryos (13–14 days) according to the Carnegie criterions [21]. The limited access to the Carnegie collection makes that no other specimens have been examined for the presence of a *notochordal process* in stage 7 (15–17 days).

In stage 8 (17–19 days) the *notochordal process* appears as an accumulation of cells in an epithelial configuration cranial to the area of gastrulation. The *notochordal process* is ventrolaterally attached to the endoderm. Based on the histologically stained sections, continuation of the endoderm ventral to the *notochordal proc*ess appears absent but cannot be excluded. A luminal structure travels ventrodorsally through the *notochordal process* between yolk sac cavity and amniotic cavity, with a clear epithelial lining ending in the primitive pit. This indisputable lumen is the *neurenteric canal* (Fig 2).

In the early stage 8 (17–19 days) embryos, the developing notochord becomes broader and thicker cranially to the *notochordal process*. It acquires a mesenchymal structure rather than an epithelial configuration (Fig 2). This structure is the *prechordal plate*, which merely is a loose configuration of mesenchymal cells. There is no single layered epithelial notochordal structure between the *notochordal process* and the *prechordal plate* present in specimen number No. 8671. Such a structure, the *notochordal plate*, however, is present to a great extent in the late stage 8 specimen numbers 10157 and 5960 [29].

The boundaries between the area of gastrulation, the *notochordal process*, *notochordal plate* and *prechordal plate* along the craniocaudal axis are difficult to distinguish in individual histological sections. However, these morphological structures can be more easily recognized and distinguished from each other in 3D reconstructions (Fig 2).

### Lengthening and bending of the developing notochord: The notochordal plate

The *notochordal process* gradually transforms into the *notochordal plate* along the axial midline in stages 9 to 10 (19–23 days), except for the most caudal part where gastrulation is still ongoing and the *notochordal process* is still present. The *notochordal process* is a transient structure and does never encompass the entire length of the developing notochord. In stages 9 to 10 (19–23 days) the *notochordal plate* is a one-cell layer thick concave structure embedded in the roof of the forming gut, which adheres tightly to the forming neural tube. In stage 9 (19–21 days) it follows the round contours of the dorsally forming neural tube along the cranial-caudal axis, by which the lateral margins of the *notochordal plate* point into dorsal direction relative to the axial midline. The whole developing notochord is now intercalated within the roof of the gut; there is no endodermal layer present ventral to the *notochordal plate* (Fig 3).

As opposed to stage 9 (19–21 days), the lateral margins of the *notochordal plate* in stage 10 (21–23 days) have folded into ventral direction relative to the axial midline. By this process the so-called notochordal ridges are formed and the lumen of the forming notochord protrudes entirely into dorsal direction, thereby acquiring an inverted U-shape (Fig 3B and 3C). Thus, whereas in stage 9 (19–21 days) the entire *notochordal plate* followed the contours of the developing neural tube, in stage 10 (19–23 days) it has assumed the form of an inverted U-shape in the middle region. However, in both cranial and caudal extremes, it still follows the contours of the developing neural tube (Fig 3B).

### The formation of the definitive notochord

In stage 11 (23–26 days) a *notochordal plate* has formed along the entire cranio-caudal axis. In the middle region of the embryo the notochordal ridges have closely approached one another ventrally, forming the *definitive notochord*. At the cranial and caudal margins of the definitive notochord, the *notochordal plate* is narrower compared to its cranial and caudal extremes and it has the typical inverted U-shape as previously described. Although a lumen can be seen in some histological sections, there seems to be no epithelial lining (Fig 3C).

After its formation and detachment from the endoderm, the *definitive notochord* becomes fully incorporated into the mesodermal mesenchyme, which enables it to migrate dorsally, away from the roof of the gut, to preserve its intimate association with the floor of the neural tube. This process proceeds from the middle of the embryo into both cranial and caudal direction. In late stage 11 (26 days) the definitive notochord has formed in the caudal and middle region, still leaving a *notochordal plate* at the most cranial end (Fig 3C). At stage 12 (26–30 days) the *notochordal plate* has fully transformed into the *definitive notochord*. In the region of the caudal neuropore, where secondary neurulation is in progress, the notochord is substantially larger in diameter and contains more cell nuclei, compared to the cranial part (Fig 3D). The notochord is now fully incorporated within the mesenchyme and is almost completely detached from the endoderm. Cranially, however, it remains in close contact with the oropharynx and caudally in contact with the hindgut. In this stage the notochord is cranially, but not caudally, detached from the neural tube (Fig 3D).

### Fusion of the paired dorsal aortae in relation to the developing notochord

**Fig 4:**
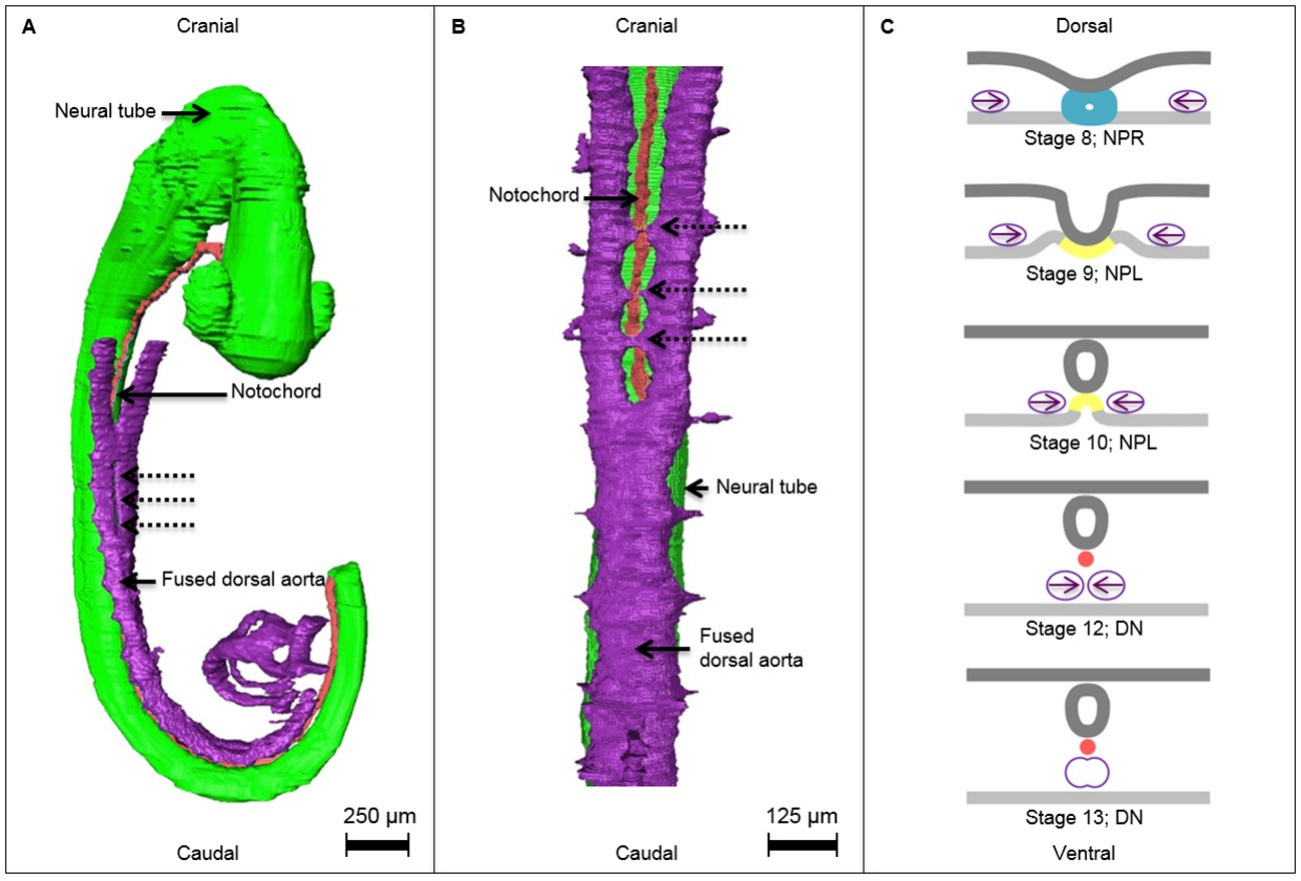
Overview of the developing notochord in relation to the fusion of the dorsal aortae. **A** 3D reconstruction of a stage 12 human embryo (26–30 days) specimen No. 8505A with ventrolateral view of the neural tube (green), the notochord (red) and dorsal aortae (purple) in the process of fusion. A part of the dorsal aorta is not fused at the dotted arrows, but cranial and caudal to this region the dorsal paired aortae are already fused. **B** A ventral close-up of the fusion process in stage 13 (28–32 days) human embryo specimen No. 836, which occurs in a ladder-like pattern. Small connections between the dorsal aortae can be appreciated (dotted arrows). **C** Transversal illustrations of the fusion of the paired dorsal aortae in relation to the developing notochord (ectoderm and is superior in each transversal section). During stages 8 to 12 (17–30 days) the paired dorsal aortae migrate from lateral to medial and fuse after the developing notochord is separated from the endoderm. Dorsal aorta: purple, *definitive notochord* (DN): red, neural ectoderm: green, *notochordal plate* (NPL): yellow, *notochordal process* (NPR): cyan blue.

The paired dorsal aortae can fuse when the notochord, which is intimately associated with the floor of the neural tube, has separated from the roof of the gut. At stage 12 (26–30 days) the fusion of the paired dorsal aortae is first observed in the midline ventral to the neural tube and notochord, and dorsal of the gut. At stage 14 (31–35 days) the paired dorsal aortae are completely fused over their entire length, except for the most caudal part where they will constitute the common iliac arteries. The locations of fusion of the dorsal aortae per studied specimen are noted in Table 2. Between these stages the notochord becomes embedded further dorsally in the mesenchyme thus creating space for the dorsal aortae to fuse. In adjacent stages, intervals of the fusing aortae were observed, causing a ladder-like pattern. Several small connections can be appreciated between the dorsal aortae, just before they fully merge (Fig 4B).

**Table 2:** Overview of sections in which the dorsal aortae are fused.

CS	Specimen #	Total number of sections	Z-res (μm)	Section numbers with fused dorsal aortae
11	6344	137	18.83	No
11	6784	577	8.70	No
12	8505A	277	10.32	126–142 144 168 = >
12	8943	436	8.22	305–357 361–380
13	836	247	16.55	122–196
13	5541	379	10.76	139–140 153 162–166 180 = >
14	6502	1107	5.01	541 = >
14	8314	639	8.07	312 338 = >

CS: Carnegie stage, Z-res: Calculated Z-resolution in μm, = >: Dorsal aortae are fused further caudally

## Discussion

**Fig 5:**
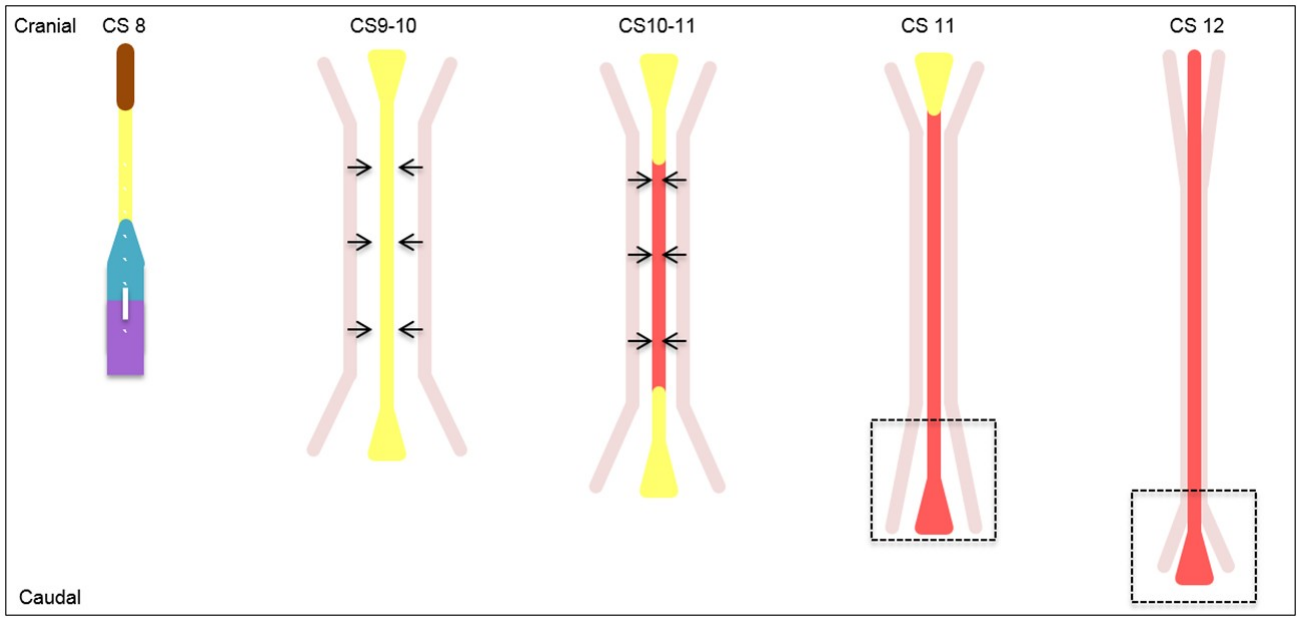
Schematic dorsal overview of the developing notochord and the fusion of the paired dorsal aortae in humans. In CS8 (17–19d.) the *notochordal process* is subsequent to the gastrulation area in direct contact with the *prechordal plate*. During CS8 to CS10 (17-23d.) the *notochordal plate* is formed between de *notochordal process* and the *prechordal plate*. Along with the axial lengthening of the whole embryo, the *notochordal plate* also lengthens, in contrast to the *notochordal process*. In CS10 to CS11 (21-26d.) the *notochordal plate* transformed into the *definitive notochord*, starting in the middle into both cranial and caudal direction. The cranial and caudal parts (triangular shaped) of the *notochordal plate* and *definitive notochord* are larger compared to the middle regions. In CS11 (23-26d.) the *notochordal plate* is cranially still present, in contrast to the caudal eminence (dotted box), where the definitive notochord is already formed due to the process of direct mesenchymal condensation. In this region the notochord is broader than in the middle and cranial region and contains more nuclei (Fig 3D). *Definitive notochord*: red, gastrulation: dark purple, *neurenteric canal*: white, *notochordal plate*: yellow, *notochordal process*: cyan blue, paired dorsal aortae: pink.

Our analysis and 3D reconstructions of 27 sectioned human embryos from Carnegie stage 7 to 15 (15–41 days), resulted in a comprehensive description of human notochordal development (Figs 2, 3 and 5).

### Human notochordal development

Notochordal development first starts at stage 7 (15–17 days) with the *notochordal process* directly rostral to the area of gastrulation. It appears as a group of undifferentiated cells that briefly persists from stage 8 to 10 (17 to 23 days) [20, 29]. Within the *notochordal process*, a connection between the amniotic cavity and yolk-sac, known as the *neurenteric canal*, is established in stages 8 to 10 (17–23 days) (Figs 2 and 3). The *neurenteric canal* has been described in several human embryos [21]. Although its existence is disputed by some [30], it was clearly present in all CS8 embryos we have studied. As we briefly mentioned previously [20], the ventral side of this cell cluster is incorporated into the endodermal roof of the gut and its dorsal side, known as the primitive pit [31], is closely attached to the developing neural tube. The *notochordal process* then incorporates entirely into the endoderm, forming the epithelial *notochordal plate* in stages 8 to 11 (17–26 days). The *notochordal plate* then acquires an “inverted U-shape” in stages 10 to 11 (21 to 26 days), relatively lengthens during these stages and remains intimately associated with the neural tube until stage 12 (26–30 days).

Subsequently, the *notochordal plate* detaches completely from the endoderm to form the definitive notochord in stage 12 (26–30 days), allowing the paired dorsal aortae to fuse in-between the notochord and the roof of the gut from stage 12 to 14 (26 to 35 days).

### Craniocaudal gradient of development

Similar to gastrulation, the formation of the *prechordal plate*, *notochordal process* and *-plate* proceeds in craniocaudal direction. However, in contrast to the mechanism described in all modern textbooks, the formation of the definitive notochord including the initial notochordal plate's acquisition of an inverted U-shape, starts in the middle of the embryo, and then proceeds in both cranial and caudal directions, similar to the closure of the neural tube [32] and the subsequent formation of vertebral neural arches [33]. In contrast to the development of the notochord as illustrated in textbooks, the human notochord does not have a stage in which the process of gastrulation and the definitive notochord are simultaneously present, as can be observed for instance in chicken embryos [13–19] (Fig 5).

Tumors derived from the notochord, so-called chordomas, preferentially localize in the skull base and sacrococcygeal regions. These tumors are suggested to be entrapped remnants of the developing notochord with malignant transformation [34]. This observation is in line with our proposed bi-directional developing notochord model. It might be that notochord formation is not completed in these regions, resulting in increased susceptibility for tumor development.

**Fig 6:**
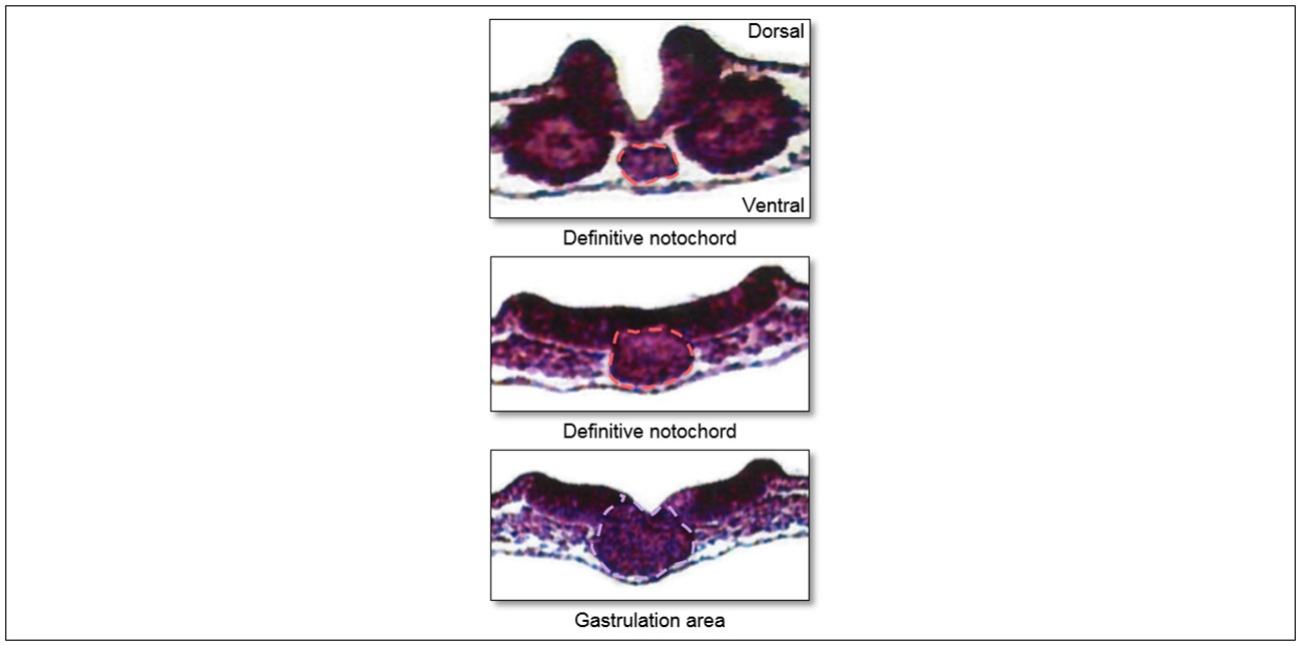
Transversal sections of the notochord in a 24 hour stage chicken embryo from our lab. Caudally, gastrulation is still on-going, while cranial, the *definitive notochord* is already present in the same stage. *Definitive notochord*: red, gastrulation: purple.

The chicken embryo displays a craniocaudal developmental gradient of the *definitive notochord*, which forms directly cranial to the area of gastrulation (Fig 6) [35, 36]. In both mice and chicken embryos a *notochordal proces*s, as seen in the human embryo, has not been observed [35, 36]. The mouse, however, does have a *notochordal plate* in the roof of the forming gut, in contrast to the chicken embryo [36]. Therefore, the notochordal plate as an intermediate to the formation of the definitive notochord, could be a mammalian synapomorphy. Further research in other mammals including Marsupial and Monotremata is necessary to substantiate this suggestion.

In mice, the developing notochord encompasses three different morphological processes along the craniocaudal axis; the anterior mesendoderm (containing both the *prechordal plate* and *anterior head process*), the *trunk notochord* and *tail notochord* [37]. In line, we have observed three different regions of morphological processes in the human developing notochord. In the cranial part, the developing notochord is formed via mesenchymal condensation forming the *prechordal plate* which transforms into the notochordal plate. Caudal to the *prechordal plate*, the formation of the *definitive notochord* occurs via the formation of the *notochordal process* and also in the *notochordal plate*. The formation of the notochord in the caudal eminence, where the *definitive notochord* is larger in diameter compared to the cranial parts, occurs via direct mesenchymal condensation [38, 39] (Fig 5). In the rat embryo these different morphological processes also seem to occur. Anteriorly the developing notochord is formed from the *anterior head process*, which also encompasses a rudimentary entrance of the *neurenteric canal*. Caudal from the *anterior head process*, the *notochordal plate* is formed, embedded in between the endoderm and in the most caudal part the notochord is formed without intercalation in between the endoderm [40]. In all, the development of the notochord in human, rat and mice occurs via three similarly morphological processes in contrast to the chicken embryo.

### Fusion of the paired dorsal aortae

The presented model also explains, in morphological terms, the timing of fusion of the paired dorsal aortae. In the human embryo, two dorsal aortae develop dorsal to the gut and lateral to the notochord. As long as the notochord is still in contact with the floor of the neural tube and the roof of the developing gut, the dorsal aortae cannot fuse in the axial midline to become the descending aorta (Fig 4).

The secretion of the bone morphogenetic protein antagonists Noggin and Chordin by the notochord in the chick embryo inhibits fusion of the dorsal aortae [41]. Additionally, Vascular Endothelial Growth Factor (VEGF) and Sonic hedgehog are known to be expressed by the notochord and to attract the dorsal aortae towards the midline, whilst Chordin expression is downregulated [41]. The exact molecular pathways are however complex and not yet fully understood in the chick embryo.

The human paired dorsal aortae fuse in a ladder-like manner (Fig 4B). This sprouting of microvasculature between the paired dorsal aortae, just cranial to the fused dorsal aorta, suggests that sprouting precedes fusion. Interestingly, VEGF is known to stimulate the sprouting of blood vessels [41].

In conclusion, here we present a model of human notochordal development that differs significantly from the models presented in human embryology textbooks and from chicken and mice morphology. We have shown that human notochordal development proceeds in a two-step fashion, first the *notochordal process* and *notochordal plate* are formed in a craniocaudal sequence. Subsequently, the definitive notochord is first formed in the middle of the embryo, which then extends into both cranial and caudal directions and detaches from the endoderm, allowing the paired dorsal aortae to fuse.

## Supporting information

S1 Fig Histological sections of three stage 8 (17–19 days) human embryos. Specimens Nos. 10157, 5960 and 7545 of the Carnegie collection with cross-sections at the same level, matching the black lines in the ventral illustration. The *notochordal process* becomes smaller, containing less nuclei or cells, in the cranial direction in specimen 5960 and 7545. Furthermore, the notochordal process is not a perfect round structure in specimen 10157, in contrast to the two other specimens. Epiblast or ectoderm: dark blue, gastrulation area (GA): purple, *notochordal process* (NPR): cyan blue, *notochordal plate* (NPL): yellow, *neurenteric canal*: white.
(TIF)
