# Supplementary figures and images for: The development of the human notochord
Source: PLoS One. 2018 Oct 22;13(10):e0205752. doi: 10.1371/journal.pone.0205752 (PMC6197658; doi:10.1371/journal.pone.0205752)

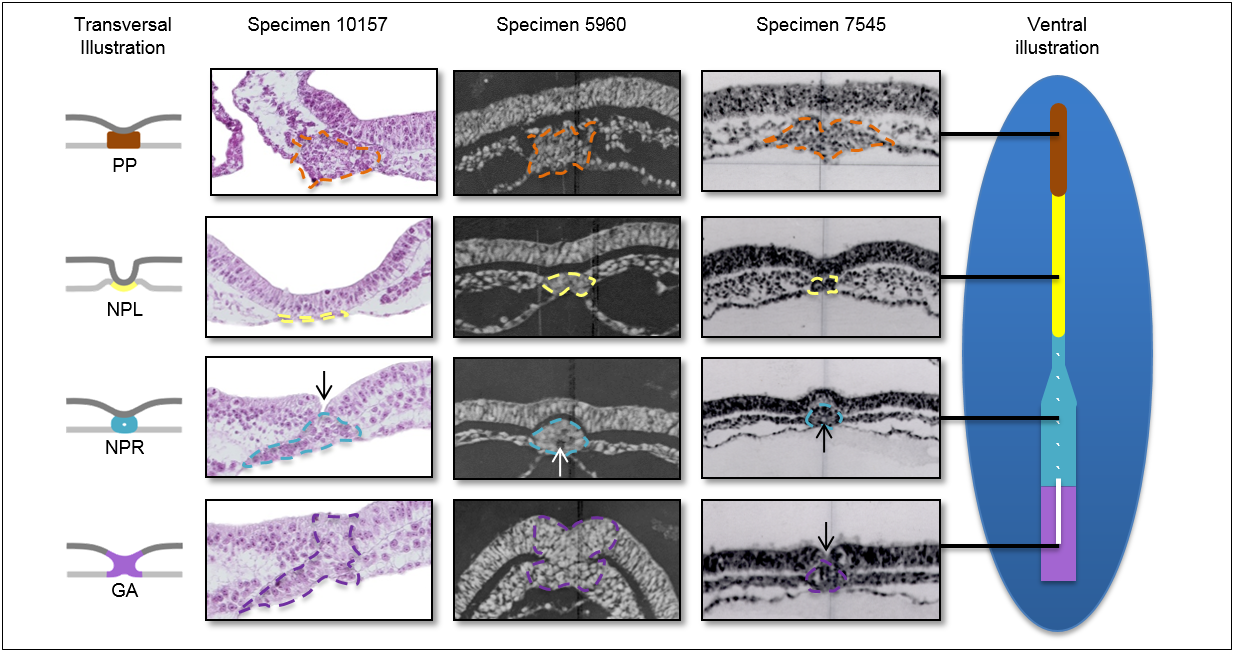

Supplement: S1 Fig — Specimens Nos. 10157, 5960 and 7545 of the Carnegie collection with cross-sections at the same level, matching the black lines in the ventral illustration. The notochordal process becomes smaller, containing less nuclei or cells, in the cranial direction in specimen 5960 and 7545. Furthermore, the notochordal process is not a perfect round structure in specimen 10157, in contrast to the two other specimens. Epiblast or ectoderm: dark blue, gastrulation area (GA): purple, notochordal process (NPR): cyan blue, notochordal plate (NPL): yellow, neurenteric canal: white. (TIF) [file pone.0205752.s001.tif]
